# Shotgun Lipidomics Identifies a Paired Rule for the Presence of Isomeric Ether Phospholipid Molecular Species

**DOI:** 10.1371/journal.pone.0001368

**Published:** 2007-12-26

**Authors:** Kui Yang, Zhongdan Zhao, Richard W. Gross, Xianlin Han

**Affiliations:** 1 Division of Bioorganic Chemistry and Molecular Pharmacology, Department of Internal Medicine, Washington University School of Medicine, St. Louis, Missouri, United States of America; 2 Department of Chemistry, Washington University, St. Louis, Missouri, United States of America; The Research Institute for Children, United States of America

## Abstract

**Background:**

Ether phospholipids are abundant membrane constituents present in electrically active tissues (e.g., heart and the brain) that play important roles in cellular function. Alterations of ether phospholipid molecular species contents are associated with a number of genetic disorders and human diseases.

**Methodology/Principal Findings:**

Herein, the power of shotgun lipidomics, in combination with high mass accuracy/high resolution mass spectrometry, was explored to identify a paired rule for the presence of isomeric ether phospholipid molecular species in cellular lipidomes. The rule predicts that if an ether phospholipid A′-B is present in a lipidome, its isomeric counterpart B′-A is also present (where the ′ represents an ether linkage). The biochemical basis of this rule results from the fact that the enzymes which participate in either the sequential oxidation of aliphatic alcohols to fatty acids, or the reduction of long chain fatty acids to aliphatic alcohols (metabolic precursors of ether lipid synthesis), are not entirely selective with respect to acyl chain length or degree of unsaturation. Moreover, the enzymatic selectivity for the incorporation of different aliphatic chains into the obligatory precursor of ether lipids (i.e., 1-*O*-alkyl-glycero-3-phosphate) is also limited.

**Conclusions/Significance:**

This intrinsic amplification of the number of lipid molecular species present in biological membranes predicted by this rule and demonstrated in this study greatly expands the number of ether lipid molecular species present in cellular lipidomes. Application of this rule to mass spectrometric analyses provides predictive clues to the presence of specific molecular species and greatly expands the number of identifiable and quantifiable ether lipid species present in biological samples. Through appropriate alterations in the database, use of the paired rule increases the number of identifiable metabolites in metabolic networks, thereby facilitating identification of biomarkers presaging disease states.

## Introduction

Plasmalogens are a unique subclass of phospholipids, containing an *O*-alk-1′-enyl aliphatic chain at the *sn*-1 position of glycerol. Plasmalogen molecular species are predominantly present in choline and ethanolamine glycerophospholipids [1], [2]. Plasmalogens exist, to varying degrees, essentially in all subcellular membranes and organelles from a variety of cells from virtually all mammalian tissues examined [1]. Previous studies have underscored the enrichment of plasmalogens in electrically active tissues such as heart and the brain [1], [3]–[5].

Altered plasmalogen content has been linked to a number of genetic disorders including Zellweger syndrome and Refsum disease [6] and neurodegenerative disorders (e.g., Alzheimer's disease) as well as psychiatric disorders (e.g., bipolarism) [4], [7]. The content and composition of plasmalogen molecular species are uniquely tailored to the specific functions of a particular cell type. For example, both gray and white matters of the brain contain abundant amounts of ethanolamine plasmalogen (i.e., plasmenylethanolamine, pPtdEtn) molecular species. The majority (55–60 mol%) of pPtdEtn molecular species in gray matter contain four or more double bonds in fatty acids esterified at the *sn*-2 position whereas 18∶1-18∶1 pPtdEtn represents over 85 mol% of the total pPtdEtn in white matter [4]. Myelin-enriched white matter requires plasmalogen (instead of its diacyl counterpart) to provide a sufficiently compact membrane structure for the efficient function of the axonal sheath [8]. Thus, pPtdEtn molecular species in white matter contain less unsaturated acyl constituents at the *sn*-2 position to enhance membrane packing. In contrast, pPtdEtn molecular species in gray matter contain abundant polyunsaturated acyl chains (e.g., 20∶4 and 22∶6 FA) which can efficiently promote membrane fusion and can serve as a reservoir of essential lipid signaling molecules [9]–[11]. Notably, these polyunsaturated pPtdEtn molecular species facilitate membrane fusion between synaptic vesicles and the neuronal plasma membrane, thereby allowing neurotransmitter release at synaptic terminals. Considering the importance of plasmalogen molecular species to cellular function, detailed investigations of ether lipid biosynthesis, remodeling, trafficking, and catabolism will depend heavily on the advances in lipidomics that facilitate increased identification, quantitation, and kinetics of the numerous plasmalogens molecular species present in biological samples.

Historically, plasmalogen molecular species have been separated by straight phase or cation exchange high performance liquid chromatography (HPLC) into lipid classes and subsequently analyzed by reversed phase HPLC [12]. In many cases, enzymatic hydrolysis of the head group and/or derivatization has been employed [2], [13]. The identities of the fractionated peaks were identified by gas chromatography (GC) or GC/mass spectrometric (MS) analyses after derivatization (e.g., acid methanolysis) [3], [12]. Fast atom bombardment (FAB) mass spectrometry has provided important information about the content of plasmalogen molecular species in heart subcellular fractions [3], [12]. Recently, increases in the sensitivity of detection of ions, largely due to the sensitivity inherent in electrospray ionization (ESI)/MS, have greatly improved the identification efficiency of low abundance plasmalogen molecular species from complex lipid mixtures [14]–[17]. However, detailed analysis of low abundance plasmalogen molecular species is still complicated by their lability and the difficulties inherent in the identification of extremely low abundance ions in general. This is particularly prominent in the case of plasmalogens that are potentially isomeric with *O*-alkyl (i.e., plasmanyl) phospholipid molecular species.

**Figure 1:**
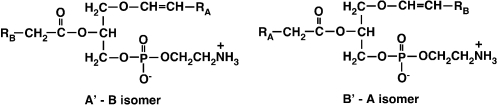
The structures of the paired isomers of plasmenylethanolamine molecular species.

During our global analyses of a variety of cellular lipid extracts using the shotgun lipidomics technology we have recently developed [18], [19], an intriguing phenomenon has been observed. Specifically, we observed a molecular ion peak corresponding to a plasmalogen molecular species in which a low abundance fatty acyl carboxylate fragment A co-exists with an abundant fatty acyl carboxylate fragment B at the identical *m/z* during precursor ion scanning or product ion analysis. This plasmalogen species contained a fatty acyl moiety B and an *O*-alk-1′-enyl aliphatic chain A′ which possessed an identical number of carbon atoms and degree of unsaturation (not including the *O*-vinyl bond) as that of carboxylate fragment A shown in Figure 1. Therefore, we hypothesized that an isomeric plasmalogen molecular species B′-A is also likely present albeit at a much lower abundance, in addition to the readily observable abundant plasmalogen molecular species A′-B (defined hereafter as “a paired rule”) (Figure 1). In this study, we exploited recent advances in high mass accuracy/high resolution mass spectrometry to examine this hypothesis with a variety of cellular lipid extracts in tissue samples from multiple organs (e.g., heart, brain, intestine, liver, kidney, and dorsal root ganglia) obtained from multiple animal species (e.g., rat, mouse, rabbit, and bovine). The results demonstrate that this “paired rule” is present for very low abundance plasmalogen molecular species as well as plasmanyl glycerophospholipid molecular species in all cells and species examined. Thus, through scanning all potentially naturally occurring fatty acyl carboxylates in a two-dimensional MS approach [19], [20], use of this rule can increase penetrance into the lipidome containing ether phospholipids.

## Materials and Methods

### Materials

Synthetic 18∶0-20∶4 plasmenylethanolamine (pPtdEtn) and other synthetic phospholipids used in the study were purchased from Avanti Polar Lipids, Inc. (Alabaster, AL, USA). Solvents for sample preparation and for MS analysis were obtained from Burdick and Jackson (Burdick and Jackson, Muskegon, MI, USA). All other chemical reagents were at least analytical grade or the best grade available and obtained from either Thermo Fisher Scientific (Pittsburgh, PA, USA) or Sigma-Aldrich Chemical Company (St. Louis, MO, USA) or as indicated.

### Preparation of lipid extracts from biological samples

Fresh bovine heart was obtained from a local slaughter house. Male mice (C57BL/6, 4 months of age) or male Sprague-Dawley rats (2 months of age) were purchased from The Jackson Laboratory (Bar Harbor, ME, USA) or Charles River Laboratories, Inc. (Wilmington, MA, USA), respectively. Male New Zealand white rabbits (2–3 lbs. body weight) were purchased from Myrtles Rabbitry, Inc. (Thompson Station, TN, USA). Animals were sacrificed by asphyxiation with carbon dioxide. Brain, heart, kidney, liver, intestine, dorsal root ganglial tissues were dissected, perfused with phosphate-buffered saline to remove blood, blotted with Kim-wipes to remove excess buffer, and immediately freeze-clamped at the temperature of liquid nitrogen. Wafers were pulverized into a fine powder with a stainless steel mortar and pestle. Each individual tissue sample (approximately 10 mg of fine powder) was weighed into a disposable glass test tube. A mixture of necessary internal standards were added to each individual homogenate prior to extraction by using a modified Bligh and Dyer procedure [21] as described previously [22]. Each lipid extract was reconstituted with a volume of 500 µl chloroform/methanol (1∶1, v/v) per mg protein. The lipid extracts were finally flushed with nitrogen, capped, and stored at −20°C for ESI/MS analyses (typically within one week). PtdEtn from bovine heart was isolated by using HPLC of lipid extracts employing a silica based cation-exchange column as previously described [23]. Each lipid solution was diluted appropriately prior to infusion and lipid analysis to prevent aggregation and minimize the residual salt content.

### Instrumentation and mass spectrometry

Lipid extracts were diluted in chloroform/methanol/isopropanol (1∶2∶4, v/v/v) to a final concentration of 10–50 pmol total lipids/µl followed by addition of a small amount of LiOH prior to direct infusion. Mass spectrometric analysis was performed on an LTQ-Orbitrap mass spectrometer (Thermo Fisher Scientific, Inc., San Jose, CA, USA) equipped with an automated nanospray apparatus (i.e., Nanomate HD, Advion Bioscience Ltd., Ithaca, NY, USA). An ionization voltage of 1.1 kV and a gas pressure of 0.3 psi on the Nanomate apparatus were employed in the analyses. The Nanomate was controlled by Chipsoft 6.3.2 software. The LTQ-Orbitrap mass spectrometer was operated in the negative ion mode under a resolution setting of 60,000. The temperature of the ion transfer tube in the ion source was set at 160°C. Product-ion analyses were conducted using a peak width setting of 1 Th by selection of the molecular ion in the linear ion trap, a normalized collision energy in the C-trap of 55%, and a gas pressure of 1 mT prior to analyses of the resultant product ions in the Orbitrap. Mass spectra in both full scan and MS/MS scan modes were acquired under the control of Xcalibur software.

Shotgun lipidomics analyses of PtdEtn molecular species of all lipid extracts were also performed on a QqQ mass spectrometer (Thermo Fisher TSQ Quantum Ultra Plus, San Jose, CA, USA) equipped with an electrospray ion source as previously described [20]. All ESI/MS analyses of lipids were conducted by direct infusion employing a Harvard syringe pump at a flow rate of 4 µl/min. Typically, a 1-min period of signal averaging was employed for each mass spectrum and a 2-min period of signal averaging was employed for each tandem MS spectrum. A mass resolution of 0.7 Th at half peak width was used for both MS and tandem MS analyses performed by the QqQ mass spectrometer. For tandem mass spectrometry, a collision gas pressure was set at 1.0 mT, but the collision energy varied with the classes of lipids analyzed as described previously [18].

### Miscellaneous

Protein concentration was determined with a bicinchroninic acid protein assay kit (Pierce, Rockford, IL, USA) using bovine serum albumin as a standard. Data from biological samples were normalized to the protein content and all data are presented as the mean±SD of n≥4 for lipid analyses.

## Results

### High mass accuracy and CID fragmentation analyses reveal the presence of paired, isomeric plasmenylethanolamine molecular species in isolated bovine heart PtdEtn

**Figure 2:**
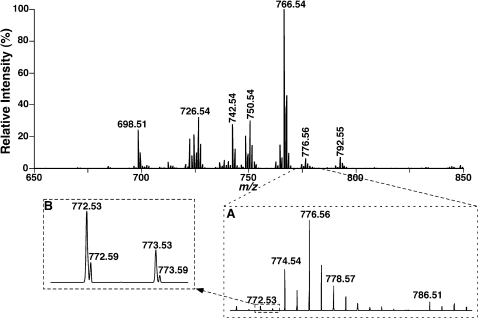
Representative negative-ion ESI/MS analyses of bovine heart ethanolamine glycerophospholipid molecular species. Bovine heart lipids were extracted by a modified Bligh and Dyer procedure [21] and the PtdEtn fraction was separated by using HPLC with a cation-exchange column as previously described [23]. Analyses of PtdEtn molecular species were performed in the negative-ion mode by using an LTQ-Orbitrap mass spectrometer equipped with a Nanomate device as described under “MATERIALS AND METHODS”.

**Figure 3:**
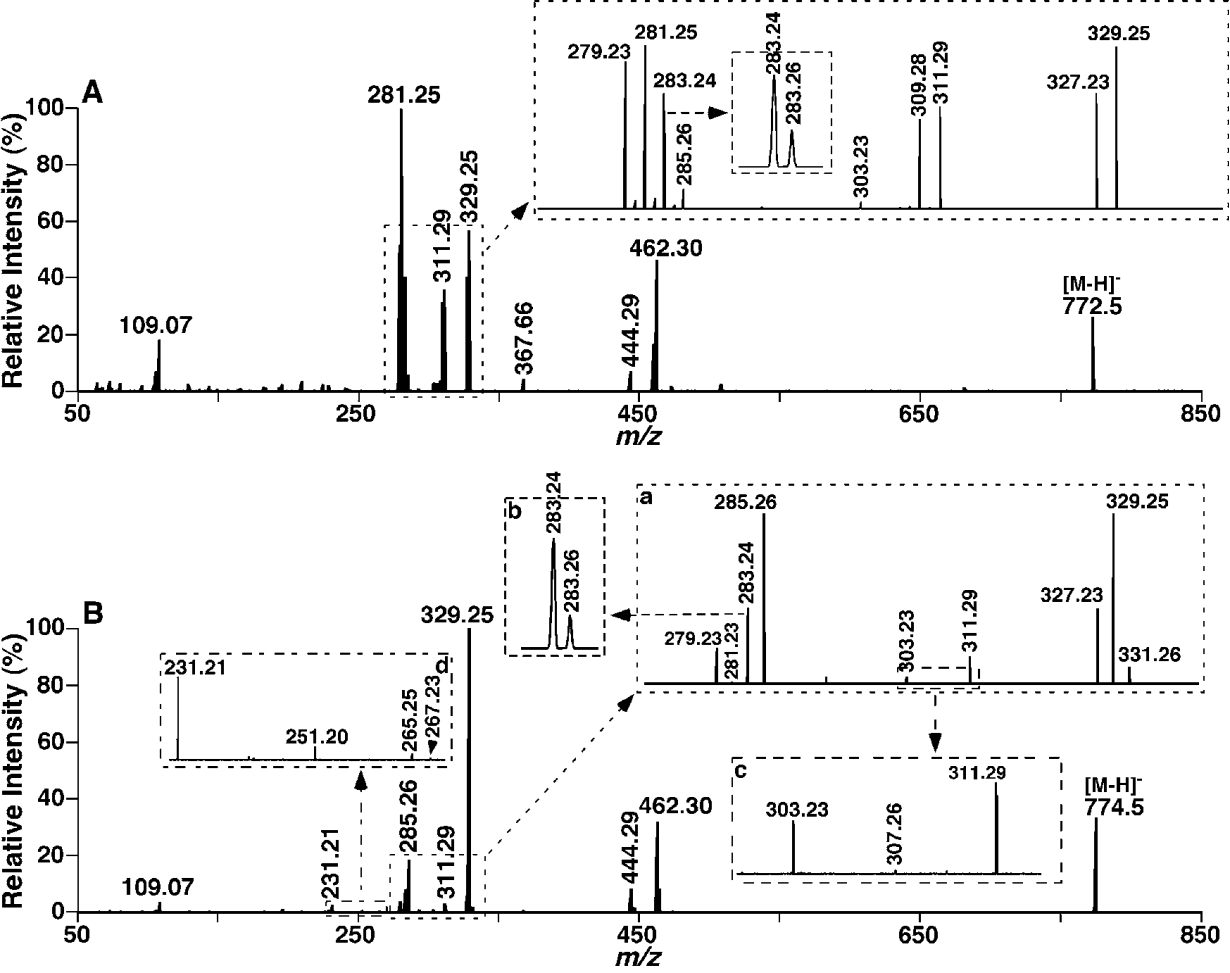
Product ion analyses of individual molecular species present in isolated bovine heart ethanolamine glycerophospholipids. Product ion ESI/MS analyses of PtdEtn molecular species in isolated bovine heart ethanolamine glycerophospholipids at *m/z* 772.5 (Panel A) and 774.5 (Panel B) were performed by using an LTQ-Orbitrap mass spectrometer. Analyses were conducted using a peak width setting of 1 Th by selection of the molecular ion in the linear ion trap (LTQ), collision activation in the C-trap with a normalized collision energy of 55% and gas pressure of 1 mTorr, and analysis of the resultant product ions in the Orbitrap.

PtdEtn molecular species were readily analyzed as deprotonated molecular ions by ESI/MS in the negative-ion mode (Figure 2) as previously described [18], [24]. Abundant pPtdEtn molecular species were present in isolated bovine heart PtdEtn as previously described [25]. However, isobaric ions to these pPtdEtn molecular species (corresponding to phosphatidylethanolamine (dPtdEtn) molecular species) were also present and were readily resolved using the high resolution present in an Orbitrap analyzer (Inset B of Figure 2). Tandem MS analysis of each individual isobaric ion peak corresponding to a representative deprotonated pPtdEtn molecular species always demonstrated the presence of multiple pPtdEtn isomers along with isomeric plasmanylethanolamine (aPtdEtn) and isobaric dPtdEtn molecular species (Figures 2 and 3 and Table 1). For example, product-ion MS analysis of an isobaric ion at *m/z* 772.5 (0.26% relative abundance to the base peak at *m/z* 766.54, Figure 2, inset A of Figure 2, and Table 1) from bovine heart PtdEtn in the negative-ion mode was performed with a mass selection window of 1 Th using an LTQ-Orbitrap mass spectrometer after collision-induced activation (CID). The CID analysis of this isobaric ion demonstrated multiple abundant fragment ions in the carboxylate region in an order of abundance at *m/z* 281.25 (i.e., 18∶1 FA), *m/z* 329.25 (i.e., 22∶5 FA), *m/z* 279.23 (i.e., 18∶2 FA), *m/z* 327.23 (i.e., 22∶6 FA), *m/z* 311.29 (i.e., 20∶0 FA), *m/z* 309.28 (i.e., 20∶1 FA), *m/z* 283.26 (i.e., 18∶0 FA), and *m/z* 303.23 (i.e., 20∶4 FA) (inset of Figure 3A). It is noteworthy that the ions at *m/z* 283.24 and 285.26 (inset of Figure 3A) correspond to aliphatic anions resulting from the loss of a CO_2_ molecule from 22∶6 FA (*m/z* 327.23) and 22∶5 FA (*m/z* 329.25), respectively. Loss of a CO_2_ molecule from polyunsaturated fatty acyl carboxylates (e.g., 20∶5, 22∶5, and 22∶6 FA) is common as previously demonstrated [5], [17]. Loss of a CO_2_ molecule from arachidonate (20∶4 FA) can also be detected, but is relatively less rapid in comparison to other aforementioned polyunsaturated fatty acids. The most common fragmentation pathway likely results from the resonance stabilized anion conjugated to olefinic π clouds. Since the content of the selected molecular ion at *m/z* 772.5 was markedly decreased in the lipid mixture following treatment with acid vapor as described previously [5], this ion largely represented a deprotonated pPtdEtn or isomeric pPtdEtn mixtures. Based on the identified FA carboxylates through accurate mass analyses, the accurate mass of the precursor ion (i.e., *m/z* 772.53), and the known information about the ion (i.e., predominantly deprotonated pPtdEtn), gave rise to the conclusion that the ion at *m/z* 772.53 is comprised of at least four pPtdEtn isomers, including 18∶2-22∶5, 18∶1-22∶6, 22∶6-18∶1, and 22∶5-18∶2 pPtdEtn molecular species in the order of relative abundance. The molar ratio of these isomers was approximately 6∶5∶5∶4 from the intensities of FA carboxylates after considering the differential fragmentation kinetics of different molecular species as previously described [24]. The relative ratios of each individual molecular species are listed in parentheses underneath the corresponding molecular species in Table 1.

**Table 1:** Identification and analyses of individual molecular species present in purified bovine heart ethanolamine glycerophospholipid^a^.

Monoisotopic mass	Molecular formula	Relative abundance (%)	pPtdEtn species^b^		aPtdEtn species^b^		dPtdEtn species^b^
			Paired species	Paired counterpart undetectable	Paired species	Paired counterpart undetectable	
694.481	p34:4/a34:5	0.43±0.03	14∶0-20∶4/20∶4-14∶0 (88.48/0.03)	16∶1-18∶3 (6.61)			
				16∶2-18∶2 (4.88)			
696.497	p34:3/a34:4	1.65±0.13	16∶1-18∶2/18∶2-16∶1 (64.76/0.02)	14∶0-20∶3 (5.9)		14∶0-20∶4 (0.51)	
			16∶0-18∶3/18∶3-16∶0 (28.81/0.01)			16∶1-18∶3 (<0.12)	
						16∶2-18∶2 (<0.09)	
698.512	p34:2/a34:3	23.49±0.51	16∶0-18∶2/18∶2-16∶0 (99.63/0.05)		16∶1-18∶2/18∶2-16∶1 (<0.37/<0.01)		
			18∶1-16∶1/16∶1-18∶1 (0.20/0.11)		16∶0-18∶3/18∶3-16∶0 (0.01/<0.01)		
712.528	p35:2/a35:3	3.82±0.29	17∶0-18∶2/18∶2-17∶0 (94.01/<0.01)				
			18∶1-17∶1/17∶1-18∶1 (0.41/0.39)				
			16∶0-19∶2/19∶2-16∶0 (0.41/0.02)				
712.492	d34:3	0.19±0.01					16∶1-18∶2/18∶2-16∶1 (4.76)
720.497	p36:5/a36:6	2.76±0.14	16∶0-20∶5/20∶5-16∶0 (62.18/0.08)	14∶0-22∶5 (4.06)			
			16∶1-20∶4/20∶4-16∶1 (30.32/0.04)				
			18∶3-18∶2/18∶2-18∶3 (3.07/0.25)				
722.512	p36:4/a36:5	17.54±0.42	16∶0-20∶4/20∶4-16∶0 (88.79/<0.01)	16∶1-20∶3 (0.99)	18∶3-18∶2/18∶2-18∶3 (<0.03/<0.01)	16∶1-20∶4 (<0.26)	
			18∶2-18∶2/18∶2-18∶2 (6.85)	14∶0-22∶4 (0.1)	16∶0-20∶5/20∶5-16∶0 (0.54/<0.01)		
			18∶1-18∶3/18∶3-18∶1 (2.72/0.01)				
724.528	p36:3/a36:4	18.71±0.82	18∶1-18∶2/18∶2-18∶1 (84.33/0.14)		16∶0-20∶4/20∶4-16∶0 (1.7/<0.01)	16∶1-20∶3 (<0.04)	
			16∶0-20∶3/20∶3-16∶0 (11.45/<0.01)		18∶2-18∶2/18∶2-18∶2 (<0.31)		
			18∶0-18∶3/18∶3-18∶0 (2.38/<0.01)		18∶1-18∶3/18∶3-18∶1 (<0.12/<0.01)		
726.544	p36:2/a36:3	27.93±1.12	18∶0-18∶2/18∶2-18∶0 (>93.87/<0.01)		18∶1-18∶2/18∶2-18∶1 (<4.76/<0.01)	18∶0-18∶3 (0.03)	
			18∶1-18∶1/18∶1-18∶1 (0.71)				
			16∶0-20∶2/20∶2-16∶0 (0.29/<0.01)		16∶0-20∶3/20∶3-16∶0 (0.32/<0.01)		
726.507	d35:3	0.003±0.000					17∶1-18∶2/18∶2-17∶1 (0.01)
736.528	p37:4/a37:5	3.27±0.23	17∶0-20∶4/20∶4-17∶0 (85.36/<0.01)	15∶0-22∶4 (1.42)			
736.492	d36:5	0.50±0.03					16∶0-20∶5/20∶5-16∶0 (12.86)
							16∶1-20∶4/20∶4-16∶1 (0.35)
738.544	p37:3/a37:4	0.31±0.04		17∶0-20∶3 (1.98)		17∶0-20∶4 (4.03)	
738.507	d36:4	4.88±0.06					18∶2-18∶2 (51.56)
							16∶0-20∶4/20∶4-16∶0 (42.43)
740.559	p37:2/a37:3	0.15±0.02	18∶0-19∶2/19∶2-18∶0 (0.07/<0.01)			17∶0-20∶3 (3.7)	
740.523	d36:3	3.83±0.10					18∶1-18∶2/18∶2-18∶1 (92.30)
							18∶0-18∶3/18∶3-18∶0 (3.91)
742.539	d36:2	24.49±0.77					18∶0-18∶2/18∶2-18∶0 (97.34)
							18∶1-18∶1 (2.66)
746.512	p38:6/a38:7	2.15±0.13	18∶1-20∶5/20∶5-18∶1 (60.23/5.59)				
			18∶2-20∶4/20∶4-18∶2 (22.15/2.38)				
			16∶1-22∶5/22∶5-16∶1 (5.5/0.02)				
			16∶0-22∶6/22∶6-16∶0 (4.01/0.12)				
748.528	p38:5/a38:6	18.98±0.46	18∶1-20∶4/20∶4-18∶1 (56.72/<0.01)		18∶1-20∶5/20∶5-18∶1 (<0.68/<0.01)		
			16∶0-22∶5/22∶5-16∶0 (25.84/0.01)		18∶2-20∶4/20∶4-18∶2 (<0.25/<0.01)		
			18∶0-20∶5/20∶5-18∶0 (16.52/0.05)		16∶1-22∶5/22∶5-16∶1 (<0.06/<0.01)		
			18∶2-20∶3/20∶3-18∶2 (0.69/<0.01)				
			16∶1-22∶4/22∶4-16∶1 (0.16/<0.01)				
750.544	p38:4/a38:5	25.98±1.29	18∶0-20∶4/20∶4-18∶0 (>76.57/0.01)		18∶1-20∶4/20∶4-18∶1 (<4.75/<0.01)	18∶0-20∶5 (0.53)	
			18∶1-20∶3/20∶3-18∶1 (9.16/<0.01)		16∶0-22∶5/22∶5-16∶0 (1.17/<0.01)		
			16∶0-22∶4/22∶4-16∶0 (6.55/0.01)		18∶2-20∶3/20∶3-18∶2 (<0.06/0.03)		
					16∶1-22∶4/22∶4-16∶1 (<0.01/<0.01)		
750.507	d37:5	0.32±0.12					17∶0-20∶5/20∶5-17∶0 (0.92)
							17∶1-20∶4/20∶4-17∶1 (0.31)
752.559	p38:3/a38:4	1.01±0.18	18∶0-20∶3/20∶3-18∶0 (>41.17/0.01)	18∶1-20∶2 (0.85)	18∶0-20∶4/20∶4-18∶0 (>3.21/<0.01)	18∶1-20∶3 (<2.26)	
						16∶0-22∶4 (1.12)	
752.523	d37:4	1.06±0.11					17∶0-20∶4/20∶4-17∶0 (51.38)
762.544	p39:5/a39:6	0.80±0.10	17∶0-22∶5/22∶5-17∶0 (16.89/0.01)				
762.507	d38:6	3.94±0.11					18∶2-20∶4/20∶4-18∶2 (77.88)
							18∶1-20∶5/20∶5-18∶1 (5.22)
764.559	p39:4/a39:5	0.34±0.04	17∶0-22∶4/22∶4-17∶0 (1.03/0.01)		17∶0-22∶5/22∶5-17∶0 (1.40/<0.01)		
764.523	d38:5	13.51±0.24					18∶0-20∶5/20∶5-18∶0 (52.77)
							18∶1-20∶4/20∶4-18∶1 (44.31)
							16∶0-22∶5/22∶5-16∶0 (0.48)
766.575	p39:3/a39:4	0.65±0.39		19∶0-20∶3 (0.64)			
766.539	d38:4	100.00					18∶0-20∶4/20∶4-18∶0 (99.36)
770.512	p40:8/a40:9	0.0005±0.0000	20∶4-20∶4/20∶4-20∶4 (32.10)				
770.570	d38:2	0.001±0.000					18∶0-20∶2/20∶2-18∶0 (34.21)
							18∶1-20∶1/20∶1-18∶1 (28.62)
							18∶2-20∶0/20∶0-18∶2 (5.08)
772.528	p40:7/a40:8	0.26±0.03	18∶2-22∶5/22∶5-18∶2 (27.15/16.02	20∶3-20∶4 (0.60)	20∶4-20∶4/20∶4-20∶4 (<0.02)		
			18∶1-22∶6/22∶6-18∶1 (19.33/19.14)				
772.586	d38:1	0.06±0.01					18∶1-20∶0/20∶0-18∶1 (9.78)
							18∶0-20∶1/20∶1-18∶0 (7.96)
774.544	p40:6/a40:7	2.60±0.22	18∶1-22∶5/22∶5-18∶1 (87.08/0.09)		18∶2-22∶5/22∶5-18∶2 (<0.53/<0.31)	20∶3-20∶4 (<0.07)	
			18∶0-22∶6/22∶6-18∶0 (7.13/0.88)		18∶1-22∶6/22∶6-18∶1 (<0.38/<0.09)		
			22∶4-18∶2/18∶2-22∶4 (2.40/1.56)				
			20∶2-20∶4/20∶4-20∶2 (0.50/< 0.01)				
774.507	d39:7	0.01±0.00					17∶1-22∶6/22∶6-17∶1 (0.27)
774.601	d38:0	0.0005±0.0000					18∶0-20∶0/20∶0-18∶0 (0.02)
776.559	p40:5/a40:6	5.65±0.35	18∶0-22∶5/22∶5-18∶0 (>82.78/0.02)	20∶1-20∶4 (1.11)	18∶1-22∶5/22∶5-18∶1 (<2.37/<0.01)	20∶2-20∶4 (<0.02)	
			18∶1-22∶4/22∶4-18∶1 (11.76/0.01)	20∶0-20∶5 (0.36)	18∶0-22∶6/22∶6-18∶0 (0.2/<0.02)		
				20∶2-20∶3 (0.02)	22∶4-18∶2/18∶2-22∶4 (0.04/<0.04)		
776.523	d39:6	0.08±0.01					19∶1-20∶5/20∶5-19∶1 (0.63)
							17∶0-22∶6/22∶6-17∶0 (0.37)
							18∶2-21∶4/21∶4-18∶2 (0.33)
778.575	p40:4/a40:5	0.92±0.11	18∶0-22∶4/22∶4-18∶0 (>61.01/0.22)	20∶0-20∶4 (6.83)	18∶0-22∶5/22∶5-18∶0 (11.88/<0.01)	18∶1-22∶4 (<1.98)	
				20∶1-20∶3 (0.69)		20∶1-20∶4 (<0.19)	
				18∶1-22∶3 (0.51)		20∶0-20∶5 (<0.31)	
						20∶2-20∶3 (<0.01)	
778.539	d39:5	0.18±0.02					17∶0-22∶5/22∶5-17∶0 (9.71)
							19∶1-20∶4/20∶4-19∶1 (4.14)
							19∶0-20∶5/20∶5-19∶0 (2.71)
782.606	p40:2/a40:3	0.001±0.000	22∶2-18∶0/18∶0-22∶2 (21.34/1.89)	22∶0-18∶2 (21.35)			
782.570	d39:3	0.001±0.000					21∶3-18∶0/18∶0-21∶3 (42.39)
							19∶0-20∶3/20∶3-19∶0 (13.04)
790.575	p41:5/a41:6	0.05±0.01		19∶0-22∶5 (2.80)			
790.539	d40:6	1.74±0.15					18∶0-22∶6/22∶6-18∶0 (46.06)
							18∶1-22∶5/22∶5-18∶1 (40.81)
							18∶2-22∶4/22∶4-18∶2 (10.06)
							20∶2-20∶4/20∶4-20∶2 (0.28)
792.591	p41:4/a41:5	0.03±0.01		19∶0-22∶4 (0.37)			
792.554	d40:5	6.81±0.31					18∶0-22∶5/22∶5-18∶0 (96.24)
							18∶1-22∶4/22∶4-18∶1 (2.44)
							20∶1-20∶4/20∶4-20∶1 (0.95)
794.606	p41:3/a41:4	0.01±0.01		21∶0-20∶3 (0.76)			
794.570	d40:4	0.86±0.13					18∶0-22∶4/22∶4-18∶0 (93.74)
							20∶0-20∶4/20∶4-20∶0 (5.15)
							18∶1-22∶3/22∶3-18∶1 (0.25)
							20∶1-20∶3/20∶3-20∶1 (0.11)
804.591	p42:5/a42:6	0.09±0.02	18∶0-24∶5/24∶5-18∶0 (30.57/1.54)	20∶0-22∶5 (42.29)			
			18∶1-24∶4/24∶4-18∶1 (6.89/0.23)	22∶1-20∶4 (12.13)			
			20∶1-22∶4/22∶4-20∶1 (5.93/0.02)				
804.554	d41:6	0.01±0.00					19∶1-22∶5/22∶5-19∶1 (10.88)
806.606	p42:4/a42:5	0.03±0.01	22∶0-20∶4/20∶4-22∶0 (14.72/0.01)	20∶0-22∶4 (7.84)	18∶0-24∶5/24∶5-18∶0 (0.06/<0.01)	20∶1-22∶4 (<0.31)	
			18∶0-24∶4/24∶4-18∶0 (7.42/3)	22∶1-20∶3 (1.22)		20∶0-22∶5 (2.65)	
				24∶2-18∶2 (5.34)			
806.570	d41:5	0.04±0.01					19∶0-22∶5/22∶5-19∶0 (57.36)
							19∶1-22∶4/22∶4-19∶1 (0.4)
Number of species in each subclass			53 (pairs)	26	26 (pairs)	19	49 (pairs)
% of total ether PtdEtn and dPtdEtn			49.55				50.45

a Bovine heart lipids were extracted by a modified Bligh and Dyer procedure [21] and the ethanolamine phospholipid (PtdEtn) fraction was separated by using HPLC as previously described [23]. Analyses of PtdEtn molecular species were performed in the negative-ion mode by using an LTQ-Orbitrap mass spectrometer with an electrospray ion source. The determined monoisotopic masses (column 1) of PtdEtn molecular species were externally calibrated relative to the base peak. The molecular formulas listed in column 2 were derived from accurate mass analyses of monoisotopic mass and were grouped into each isobaric mass. The prefix “a”, “d”, and “p” stand for alkyl-acyl PtdEtn, diacyl PtdEtn, and plasmalogen PtdEtn, respectively. The relative abundance listed in column 3 was normalized to the isobaric base peak of the ion at *m/z* 766.5 after ^13^C de-isotoping and represents X±SD of at least four different analyses. The notation m∶n represents the fatty acyl (or ether aliphatic) chain containing m carbons and n double bonds. The numbers in the parentheses represent the relative composition of each individual molecular species of an isobaric ion. The symbols of “<” and “>” indicate that the data represent the best estimation from the analyses.

b Identification of individual pPtdEtn molecular species was performed based on both accurate mass analyses and acidic vapor treatment. Identification of individual aPtdEtn molecular species was performed based on the accurate mass analyses, the paired rule, and the information of the identified pPtdEtn counterparts as discussed in the text. Identification of individual dPtdEtn molecular species was conducted solely based on accurate mass analyses. The abundance of each of the paired dPtdEtn molecular species cannot be accurately determined at the current stage of lipidomic technology.

The presence of modestly abundant fragment ions derived from lysoPtdEtn further supported the identification of these pPtdEtn molecular species. However, previously identified *O*-alkenyl ions resulting from the *O*-alk-1′-enyl chain of pPtdEtn [25] were either present at very low intensities or could not be detected under the experimental conditions employed that were largely dependent on the abundance of the corresponding molecular ions. These results indicate that pPtdEtn isomers of the examined ion in the negative-ion ESI/MS analysis of bovine heart PtdEtn occur in pairs (i.e., 18∶1-22∶6/22∶6-18∶1 and 18∶2-22∶5/22∶5-18∶2 pPtdEtn). Furthermore, 20∶4-20∶4 aPtdEtn was also identified from the presence of a very low abundance fragment ion at *m/z* 303.23 (i.e., 20∶4 FA) in the CID fragment analyses of the selected isobaric ion, which along with the presence of 20∶3-20∶4 pPtdEtn is further described below in detail. Accurate mass analyses of the fragments corresponding to carboxylates (i.e., *m/z* 281.25 (i.e., 18∶1 FA), *m/z* 311.29 (i.e., 20∶0 FA), *m/z* 309.28 (i.e., 20∶1 FA), *m/z* 283.26 (i.e., 18∶0 FA)) identified the presence of 18∶1-20∶0/20∶0-18∶1 and 18∶0-20∶1/20∶1-18∶0 dPtdEtn from the selected isobaric ion at *m/z* 772.59 (Table 1).

Next, we determined if pPtdEtn molecular species pairing was present in other pPtdEtn molecular ions having more complicated isomeric patterns. For example, the CID mass spectrum of the acid-labile pPtdEtn ion at *m/z* 774.5 from bovine heart PtdEtn in the negative-ion mode (Figure 3B, 2.60% relative abundance to the base peak at *m/z* 766.54 of the mass spectrum (Figure 2)) displays multiple fragment ions in the carboxylate region. These fragments included ions at *m/z* 329.25 (i.e., 22∶5 FA), *m/z* 285.26 (i.e., loss of a CO_2_ from 22∶5 FA), *m/z* 327.23 (i.e., 22∶6 FA), *m/z* 283.24 (i.e., loss of a CO_2_ from 22∶6 FA), *m/z* 279.23 (i.e., 18∶2 FA), *m/z* 283.26 (i.e., 18∶0 FA), *m/z* 311.29 (i.e., 20∶0 FA), *m/z* 331.26 (i.e., 22∶4 FA), *m/z* 303.23 (i.e., 20∶4 FA), *m/z* 281.23 (i.e., 18∶1 FA), *m/z* 307.26 (i.e., 20∶2 FA), *m/z* 265.25 (i.e., corresponding to 9-octadecen-1′-enolate), and *m/z* 267.23 (i.e., 17∶1 FA) in order of abundance (insets of Figure 3B). Obviously, four pairs of pPtdEtn molecular species including 18∶1-22∶5/22∶5-18∶1, 18∶0-22∶6/22∶6-18∶0, 22∶4-18∶2/18∶2-22∶4, 20∶2-20∶4/20∶4-20∶2 pPtdEtn were identified from the molecular ion at *m/z* 774.54 through accurate mass analyses of the obtained carboxylate fragments. The presence of 9-octadecen-1′-enolate results from the fact that pPtdEtn molecular species containing 18∶1 vinyl ether is the most abundant molecular ion present in comparison to others. Again, the presence of low abundance isomeric 18∶1-22∶6/22∶6-18∶1 and 18∶2-22∶5/22∶5-18∶2 aPtdEtn within the molecular ion of *m/z* 774.54 (Table 1) were also identified and are described below. Accurate mass analyses of the fragments corresponding to carboxylates also identified the presence of 17∶1-22∶6/22∶6-17∶1 dPtdEtn from the very low abundant isobaric ion at *m/z* 774.51 and the presence of 18∶0-20∶0/20∶0-18∶0 dPtdEtn from another very low abundance isobaric ion at *m/z* 774.60 (Table 1).

The paired pPtdEtn molecular species present in bovine heart PtdEtn mixtures were extensively examined through accurate mass analyses of fragments resulting from CID of all acid-labile PtdEtn ions (Table 1). The results demonstrated that pPtdEtn molecular species were paired in most of the examined ions corresponding to acid-labile peaks (i.e., pPtdEtn species) except a number of very low abundant pPtdEtn molecular species (Table 1). Collectively, a total 53 pairs of pPtdEtn molecular species were identified from purified bovine heart PtdEtn while 26 pPtdEtn molecular species were identified that had no obvious ion pairs likely due to their extremely low abundance (Table 1).

### Validation of the paired rule with synthetic lipids in conjunction with acid or base treatment

**Figure 4:**
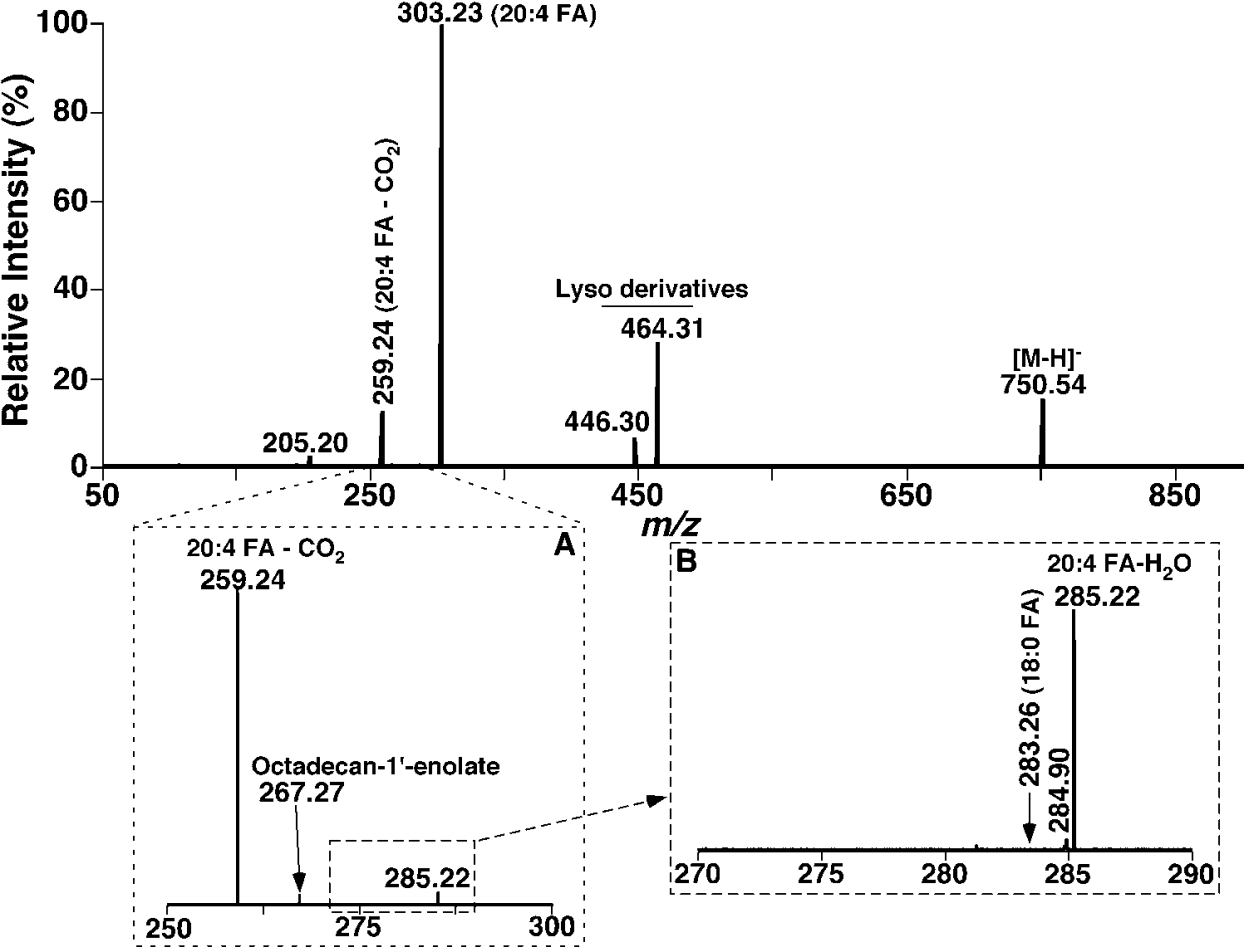
Product ion analyses of synthetic 18∶0-20∶4 plasmenylethanolamine molecular species in the negative-ion mode. Product ion ESI/MS analysis of deprotonated 18∶0-20∶4 plasmenylethanolamine at *m/z* 750.54 was performed on an LTQ-Orbitrap mass spectrometer with a C-trap using an ion selective window of 1 Th by LTQ. Collision activation in C-trap was carried out with normalized collision energy of 55% and gas pressure of 1 mT. The resultant fragment ions were analyzed in the Orbitrap. The arrow indicates the absence of the 18:0 FA carboxylate in the spectrum after amplifying the position greater than1,000 fold.

To exclude the possibility that one of the paired pPtdEtn molecular species which generates FA carboxylate (usually in low or very low abundance after CID) was due to the presence of an unknown fragmentation pathway of pPtdEtn molecular ions, additional experiments on the analyses of synthetic pPtdEtn molecular species were conducted. For example, tandem MS analysis of 18∶0-20∶4 pPtdEtn after CID demonstrates an abundant fragment ion at *m/z* 303.23 (*i.e.*, 20∶4 FA carboxylate), a modest fragment ion at *m/z* 259.24 (corresponding to the aliphatic anion resulting from the loss of a CO_2_ molecule from arachidonate as mentioned above), two fragment ions at *m/z* 446.30 and 464.31 (*i.e.*, lyso-pPtdEtn derivatives), and a very low abundance ion at *m/z* 267.27 (corresponding to octadecan-1′-enolate which indicates that the pPtdEtn molecular species containing 18∶0 vinyl ether is the most abundant one in all isomeric species) (Figure 4). No detectable fragment ion at *m/z* 283.26 (i.e., 18∶0 FA carboxylate) was present in the mass spectrum (Figure 4 inset). Identical results were also obtained from the analyses of other synthetic plasmalogen molecular species (data not shown). Accordingly, the very low abundance FA carboxylate fragments present in the tandem MS analyses of pPtdEtn in bovine heart PtdEtn did not result from an unknown CID pathway, further supporting the presence of the paired pPtdEtn molecular species in biological lipid extracts.

**Figure 5:**
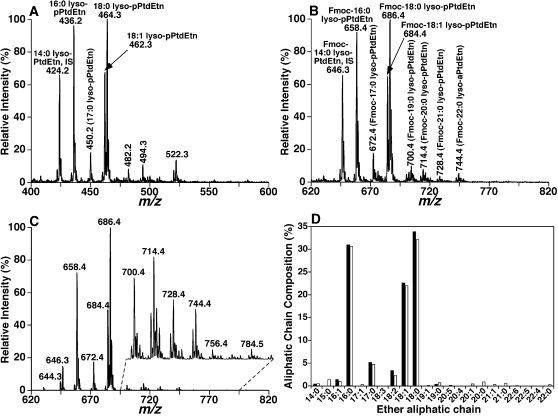
Quantitative analyses of ether lysoPtdEtn produced by alkaline hydrolysis of fatty acyl esters in bovine heart PtdEtn. Bovine heart PtdEtn solution was treated with LiOMe leading to the cleavage of the FA ester bonds in the PtdEtn pool as previously described [26]. The resultant lysoPtdEtn mixture containing an ether aliphatic chain at the *sn*-1 position of glycerol was recovered using a modified procedure of Bligh and Dyer [21]. The mass spectrum in panel A was acquired in the negative-ion mode by using a QqQ mass spectrometer directly from the lysoPtdEtn solution that was diluted to less than 50 pmol of total lipids/µl. The mass spectrum in panel B was acquired in the negative-ion mode directly from a diluted lysoPtdEtn solution after addition of Fmoc chloride as previously described [16]. The mass spectrum in panel C was acquired in the negative-ion mode as that of spectrum B but in the neutral loss mode. The neutral loss scanning was conducted through coordinately scanning the first and third quadrupoles with a mass difference of 222.2 u (i.e., loss of a Fmoc) while the collisional activation was performed in the second quadrupole at collision energy of 32 eV. “IS” denotes internal standard. Panel D shows the comparison of ether aliphatic chain profiles in bovine heart PtdEtn determined by using different approaches: (1) direct quantitation of the resultant ether lysoPtdEtn molecular species from alkaline hydrolysis of PtdEtn (closed column) through a two-step quantitation procedure [5] from spectra A and C and (2) calculated composition (open column) derived from individual PtdEtn molecular species listed in Table 1.

To further validate the paired rule for pPtdEtn molecular species, we performed additional experiments to directly demonstrate the presence of very long aliphatic chains containing high degrees of unsaturation which are usually present in one of the paired pPtdEtn molecular species with low to very low abundance (Table 1). The bovine heart PtdEtn mixture was treated with 1 M LiOMe to cleave the FA ester bonds of PtdEtn as previously described [26]. The resultant solution was washed with 1∶4 (v/v) ethyl ether/hexane three times to remove the fatty acyl esters generated by this procedure. The resultant lysoPtdEtn containing an ether aliphatic chain at the *sn*-1 position of glycerol was recovered by using a modified Bligh and Dyer extraction procedure [21]. The ether lysoPtdEtn molecular species were analyzed by ESI/MS in the negative-ion mode and the presence of very long aliphatic chains containing high degrees of unsaturation in low abundance (e.g., the ion at *m/z* 510.2) was detected (Figure 5A). These low abundance ether lysoPtdEtn molecular species were more clearly visualized after Fmoc chloride treatment (Figure 5B) as previously described [16], particularly in the mass spectrum acquired by neutral loss of the Fmoc moiety (neutral loss of 222.2 u) (Figure 5C). Quantitative analyses of these ether lysoPtdEtn molecular species in comparison to an internal standard after ^13^C deisotoping [18], [27] corresponded well with the aliphatic chain composition of the ether PtdEtn molecular species listed in Table 1 (Figure 5D). These results support the hypothesis that paired pPtdEtn isomers are present in lipid extracts of biological samples.

To confirm that the major contents of these ether-containing lysoPtdEtn molecular species contained vinyl ether linkages (and not O-alkyl ether bonds (i.e., lyso-aPtdEtn)), lysoPtdEtn resulting from alkaline hydrolysis treatment along with the internal standard (which was added during the Bligh and Dyer extraction) were further treated with acidic vapor to specifically cleave vinyl ether bonds. The recovered lyso-aPtdEtn molecular species with the lyso-dPtdEtn internal standard were analyzed by ESI/MS in the negative-ion mode. The abundance of residual lyso-aPtdEtn molecular species was not significantly above background relative to the internal standard (spectrum not shown), indicating the low abundance of aPtdEtn molecular species in bovine heart PtdEtn. The quantitative results obtained from this approach agreed well with the results obtained through accurate mass analyses (Table 1).

### Shotgun lipidomics reveals the presence of paired isomers of plasmalogen molecular species in a wide variety of lipid extracts of biological samples from multiple cell types and species

**Figure 6:**
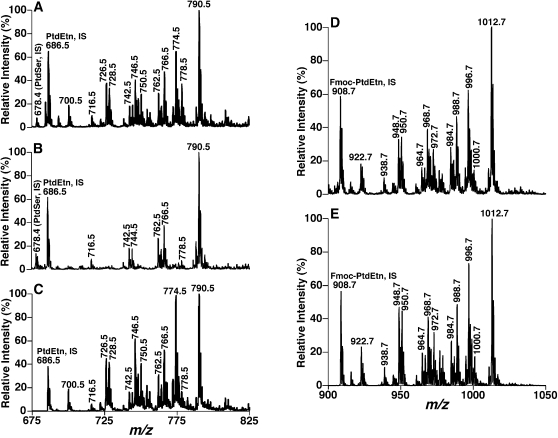
Representative negative-ion ESI/MS analyses of individual ethanolamine glycerophospholipid molecular species in mouse cerebellar lipid extracts. Mouse cerebellar lipid extracts were prepared by a modified Bligh and Dyer procedure [21]. Spectrum A was acquired in the negative-ion mode by using a QqQ mass spectrometer directly from a lipid extract that was diluted to less than 50 pmol of total lipids/µl after addition of approximately 25 pmol LiOH/µl to the lipid solution. Spectrum B was taken in the negative-ion mode after the diluted lipid solution used in spectrum A was treated with acid vapor and a small amount of LiOH (approximately 25 pmol LiOH/µl) was added to the infused solution. Spectrum C was acquired in the negative-ion mode as that of spectrum A but in the precursor-ion mode. The tandem mass spectrometry of precursor-ion scanning of 196 Th (i.e., phosphoethanolamine) was conducted through scanning the first quadrupole in the interested mass range and monitoring the third quadruple with the ion at *m/z* 196 while collision activation was performed in the second quadrupole at collision energy of 50 eV. Spectrum D was acquired in the negative-ion mode directly from a diluted mouse cerebellum lipid extract after addition of Fmoc chloride as previously described [16]. Spectrum E was acquired in the negative-ion mode as that of spectrum D but in the neutral loss mode. Tandem mass spectrometry of neutral loss scanning was conducted through coordinately scanning the first and third quadrupoles with a mass difference of 222.2 u (i.e., loss of a Fmoc) while collisional activation was performed in the second quadrupole at collision energy of 32 eV. “IS” denotes internal standard. All mass spectral traces are displayed after normalization to the base peak in each individual spectrum. All spectra are displayed after being normalized to the base peak in individual spectrum.

Next, we examined whether this paired rule was applicable to the analysis of acid-labile, plasmalogen molecular species present in lipid extracts from multiple mammalian tissues or extracellular fluid samples including brain, heart, intestine, kidney, liver, and dorsal root ganglia of rat, rabbit, mouse, and/or human. A shotgun lipidomics approach was utilized to identify and quantify lipid molecular species following intrasource separation as described previously [18]–[20]. For example, PtdEtn molecular species from mouse cerebellum were analyzed by survey scanning in the negative-ion mode using an LTQ-Orbitrap mass spectrometer after direct infusion of diluted lipid extract solutions supplemented with a small amount LiOH (similar to Figure 6A). CID analyses with accurate mass were performed to identify the paired rule of pPtdEtn molecular species.

The results from the analyses of mouse cerebellar lipid extracts were tabulated (Table 2) to include (1) all identified PtdEtn ions shown in the survey scan with their determined accurate masses (column 1), their corresponding formulas derived from their accurate masses (column 2), and their contents which were ratiometrically determined in comparison to the selected internal standard after ^13^C de-isotoping [18], [27] (column 3); (2) pPtdEtn molecular species with or without their identified paired counterparts (column 4); (3) aPtdEtn molecular species with or without their identified paired counterparts (column 5); (4) dPtdEtn molecular species identified (column 6); and (5) the composition of FA carboxylates in each isobaric peak which resulted from the fragmentation of each individual ion after CID (in the parentheses under each PtdEtn molecular species of columns 4–6). A total of 47 pairs of pPtdEtn molecular species in mouse cerebellar lipid extracts were identified while 15 pPtdEtn molecular species were also identified without pairing. These pPtdEtn species are likely unpaired due to their extremely low abundance (Table 2). The content of these 15 pPtdEtn molecular species only accounts for 0.34 mol% of total ether PtdEtn content or 0.16 mol% of total mouse cerebellar PtdEtn content (Table 2).

**Table 2:** Identification and analyses of ether-containing ethanolamine phospholipid molecular species in lipid extracts of mouse cerebellum^a^.

Monoisotopic mass	Molecular formula	Content (nmol/mg protein)	pPtdEtn species^b^		aPtdEtn species^b^		dPtdEtn species^b^
			Paired species	Paired counterpart undetectable	Paired species	Paired counterpart undetectable	
674.512	p32:0/a32:1	0.01±0.00	16∶0-16∶0/16∶0-16∶0 (100)				
686.476	d32:2 (Internal standard)	22.50±0.00					16∶1-16∶1/16∶1-16∶1 (100)
698.512	p34:2/a34:3	0.18±0.00	18∶1-16∶1/16∶1-18∶1 (72.99/5.24)				
			16∶0-18∶2/18∶2-16∶0 (17.68/4.08)				
700.528	p34:1/a34:2	3.39±0.05	16∶0-18∶1/18∶1-16∶0 (85.78/11.52)		18∶1-16∶1/16∶1-18∶1 (2.62/<0.03)		
					18∶2-16∶0/16∶0-18∶2 (<0.02/0.01)		
712.528	p35:2/a35:3	0.01±0.00	17∶1-18∶1/18∶1-17∶1 (49.16/26.32)				
			19∶1-16∶1/16∶1-19∶1 (21.75/0.05)				
			19∶2-16∶0/16∶0-19∶2 (1.59/0.23)				
712.492	d34:3	0.0001±0.0000					18∶2-16∶1/16∶1-18∶2 (0.89)
714.544	p35:1/a35:2	0.14±0.00	16∶0-19∶1/19∶1-16∶0 (>20.56/<11.65)		17∶1-18∶1/18∶1-17∶1 (<6.66/<3.56)		
					19∶1-16∶1/16∶1-19∶1 (<2.95/<0.01)		
					19∶2-16∶0/16∶0-19∶2 (<0.21/0.07)		
714.507	d34:2	0.17±0.01					16∶1-18∶1/18∶1-16∶1 (>47.73)
							16∶0-18∶2/18∶2-16∶0 (<5.96)
							17∶1-17∶1/17∶1-17∶1 (>0.63)
716.523	d34:1	1.78±0.02					16∶0-18∶1/18∶1-16∶0 (100)
722.512	p36:4/a36:5	0.96±0.02	16∶0-20∶4/20∶4-16∶0 (98.94/1.06)				
724.528	p36:3/a36:4	0.38±0.01	18∶2-18∶1/18∶1-18∶2 (53.97/13.80)		16∶0-20∶4/20∶4-16∶0 (11.36/<0.09)		
			16∶0-20∶3/20∶3-16∶0 (20.78/<0.01)				
726.544	p36:2/a36:3	10.23±0.21	18∶1-18∶1/18∶1-18∶1 (97.84)		18∶1-18∶2/18∶2-18∶1 (0.74/<0.15)		
			16∶0-20∶2/20∶2-16∶0 (1.17/<0.01)		16∶0-20∶3/20∶3-16∶0 (0.06/<0.01)		
			16∶1-20∶1/20∶1-16∶1 (0.02/0.01)				
728.559	p36:1/a36:2	5.49±0.11	18∶0-18∶1/18∶1-18∶0 (>55.13/0.39)		18∶1-18∶1/18∶1-18∶1 (<15)		
			16∶0-20∶1/20∶1-16∶0 (29.24/0.06)		16∶0-20∶2/20∶2-16∶0 (0.03/<0.01)		
					20∶1-16∶1/16∶1-20∶1 (0.16/<0.01)		
728.523	d35:2	0.0001±0.0000					17∶1-18∶1/18∶1-17∶1 (0.002)
736.528	p37:4/a37:5	0.05±0.00		19∶3-18∶1 (69.29)			
				19∶2-18∶2 (28.69)			
736.492	d36:5	0.001±0.000					16∶1-20∶4/20∶4-16∶1 (1.55)
							16∶0-20∶5/20∶5-16∶0 (0.47)
738.544	p37:3/a37:4	0.0001±0.0000	19∶2-18∶1/18∶1-19∶2 (>0.01/<0.01)	19∶1-18∶2 (<0.01)		19∶3-18∶1 (<0.01)	
738.507	d36:4	0.84±0.01					16∶0-20∶4/20∶4-16∶0 (99.99)
							18∶2-18∶2/18∶2-18∶2 (<0.01)
740.559	p37:2/a37:3	0.06±0.01	19∶1-18∶1/18∶1-19∶1 (15.40/10.27)			19∶1-18∶2 (<0.01)	
			19∶0-18∶2/18∶2-19∶0 (11.82/0.05)				
			20∶2-17∶0/17∶0-20∶2 (0.13/0.06)				
740.523	d36:3	0.10±0.05					18∶2-18∶1/18∶1-18∶2 (62.27)
							16∶0-20∶3/20∶3-16∶0 (<0.01)
742.575	p37:1/a37:2	0.001±0.000	19∶1-18∶0/18∶0-19∶1 (0.005/0.005)	17∶0-20∶1 (0.01)	19∶1-18∶1/18∶1-19∶1 (<0.001/<0.001)	17∶0-20∶2 (<0.001)	
			16∶0-21∶1/21∶1-16∶0 (0.001/<0.001)				
742.539	d36:2	4.38±0.06					18∶1-18∶1/18∶1-18∶1 (97.61)
							18∶0-18∶2/18∶2-18∶0 (2.09)
							16∶0-20∶2/20∶2-16∶0 (>0.27)
							16∶1-20∶1/20∶1-16∶1 (>0.01)
744.554	d36:1	1.57±0.01					18∶0-18∶1/18∶1-18∶0 (95.10)
							16∶0-20∶1/20∶1-16∶0 (4.90)
746.512	p38:6/a38:7	5.80±0.12	16∶0-22∶6/22∶6-16∶0 (99.43/<0.01)	18∶2-20∶4 (0.55)			
746.570	d36:0	0.001±0.000					18∶0-18∶0/18∶0-18∶0 (0.02)
748.528	p38:5/a38:6	1.88±0.07	18∶1-20∶4/20∶4-18∶1 (75.87/7.11)		16∶0-22∶6/22∶6-16∶0 (11.28/<0.01)		
			16∶0-22∶5/22∶5-16∶0 (2.93/2.80)				
750.544	p38:4/a38:5	5.08±0.12	18∶0-20∶4/20∶4-18∶0 (69.49/0.01)		18∶1-20∶4/20∶4-18∶1 (<2.34/<0.01)		
			16∶0-22∶4/22∶4-16∶0 (23.44/<0.01)		16∶0-22∶5/22∶5-16∶0 (0.04/<0.01)		
			18∶1-20∶3/20∶3-18∶1 (4.39/<0.01)				
750.507	d37:5	0.02±0.01					17∶1-20∶4/20∶4-17∶1 (0.17)
							15∶1-22∶4/22∶4-15∶1 (0.12)
752.559	p38:3/a38:4	0.0001±0.0000	18∶1-20∶2/20∶2-18∶1 (40.94/1.46)	16∶0-22∶3 (6.13)	18∶0-20∶4/20∶4-18∶0 (10.82/<0.01)	16∶0-22∶4 (10.14)	
			18∶0-20∶3/20∶3-18∶0 (>26.75/0.05)		18∶1-20∶3/20∶3-18∶1 (<3.69/<0.01)		
754.575	p38:2/a38:3	3.27±0.04	18∶1-20∶1/20∶1-18∶1 (95.33/2.08)		18∶1-20∶2/20∶2-18∶1 (2.54/<0.05)		
754.539	d37:3	0.0001±0.0000					15∶1-22∶2/22∶2-15∶1 (0.003)
756.591	p38:1/a38:2	1.37±0.02	18∶0-20∶1/20∶1-18∶0 (>73.12/<0.01)		18∶1-20∶1/20∶1-18∶1 (<12.35/<0.27)		
			18∶1-20∶0/20∶0-18∶1 (9.94/>2.36)				
			16∶0-22∶1/22∶1-16∶0 (1.94/0.01)				
756.554	d37:2	0.0001±0.0000					16∶1-21∶1/21∶1-16∶1 (0.01)
762.544	p39:5/a39:6	0.0001±0.0000	18∶1-21∶4/21∶4-18∶1 (0.001/<0.001)				
762.507	d38:6	7.58±0.07					16∶0-22∶6/22∶6-16∶0 (99.84)
							18∶2-20∶4/20∶4-18∶2 (0.14)
							18∶1-20∶5/20∶5-18∶1 (0.02)
764.559	p39:4/a39:5	0.0001±0.0000		17∶0-22∶4 (0.005)			
764.523	d38:5	2.10±0.09					18∶1-20∶4/20∶4-18∶1 (99.995)
							16∶0-22∶5/22∶5-16∶0 (<0.001)
766.575	p39:3/a39:4	0.0001±0.0000		19∶0-20∶3 (0.001)			
766.539	d38:4	9.72±0.17					18∶0-20∶4/20∶4-18∶0 (98.95)
							16∶0-22∶4/22∶4-16∶0 (1.05)
770.512	p40:8/a40:9	0.0001±0.0000	20∶4-20∶4/20∶4-20∶4 (0.02)				
770.570	d38:2	0.51±0.00					18∶1-20∶1/20∶1-18∶1 (99.98)
772.528	p40:7/a40:8	2.40±0.07	18∶1-22∶6/22∶6-18∶1 (82.79/7.75)				
772.586	d38:1	0.25±0.01					18∶0-20∶1/20∶1-18∶0 (9.46)
774.544	p40:6/a40:7	21.60±0.30	18∶0-22∶6/22∶6-18∶0 (96.03/<0.01)		18∶1-22∶6/22∶6-18∶1 (<1.80/<0.17)		
			18∶1-22∶5/22∶5-18∶1 (1.19/>0.80)				
774.601	d38:0	0.003±0.000					18∶0-20∶0/20∶0-18∶0 (<0.01)
							16∶0-22∶0/22∶0-16∶0 (<0.01)
776.559	p40:5/a40:6	0.10±0.05	18∶1-22∶4/22∶4-18∶1 (65.27/<0.01)	20∶1-20∶4 (0.15)	18∶0-22∶6/22∶6-18∶0 (9.65/<0.01)		
			18∶0-22∶5/22∶5-18∶0 (4.55/0.03)		18∶1-22∶5/22∶5-18∶1 (<0.15/<0.01)		
776.523	d39:6	0.03±0.02					17∶0-22∶6/22∶6-17∶0 (20.20)
778.575	p40:4/a40:5	1.32±0.02	18∶1-22∶3/22∶3-18∶1 (1.75/<0.01)	18∶0-22∶4 (>8.46)	18∶1-22∶4/22∶4-18∶1 (<89.79/<0.01)		
782.606	p40:2/a40:3	0.30±0.01	18∶1-22∶1/22∶1-18∶1 (92.68/0.33)				
			20∶1-20∶1/20∶1-20∶1 (5.50)				
			18∶0-22∶2/22∶2-18∶0 (>1.29/0.15)				
782.570	d39:3	0.0001±0.0000					17∶1-22∶2/22∶2-17∶1 (0.04)
784.622	p40:1/a40:2	0.08±0.00	22∶0-18∶1/18∶1-22∶0 (56.95/0.75)	18∶0-22∶1 (31.53)	18∶1-22∶1/22∶1-18∶1 (<1.42/<0.01)		
			20∶0-20∶1/20∶1-20∶0 (5.56/0.05)		20∶1-20∶1/20∶1-20∶1 (<0.08 )		
784.586	d39:2	0.003±0.000					17∶1-22∶1/22∶1-17∶1 (3.16)
784.528	p41:8/a41:9	0.0004±0.0000		19∶2-22∶6 (0.50)			
788.523	d40:7	3.10±0.07					18∶1-22∶6/22∶6-18∶1 (100)
790.539	d40:6	36.11±0.35					18∶0-22∶6/22∶6-18∶0 (100)
806.606	p42:4/a42:5	0.06±0.00		22∶0-20∶4 (35.31)			
				18∶0-24∶4 (38.25)			
				20∶0-22∶4 (26.44)			
838.539	d44:10	0.75±0.03					22∶4-22∶6/22∶6-22∶4 (100)
Number of species in each subclass			47 (pairs)	15	25 (pairs)	4	40 (pairs)
% of total ether PtdEtn and dPtdEtn			48.18				51.82

a Mouse cerebellar lipids were extracted by using a modified Bligh and Dyer procedure [21]. Analyses of PtdEtn molecular species were performed in the negative-ion mode using an LTQ-Orbitrap mass spectrometer with an electrospray ion source by using a shotgun lipidomics approach. The determined monoisotopic masses (column 1) of each PtdEtn molecular species were externally calibrated relative to the selected internal standard at *m/z* 686.476. The molecular formulas listed in column 2 were derived from accurate mass analyses of monoisotopic mass and were grouped into each isobaric mass. The prefix “a”, “d”, and “p” stand for alkyl-acyl PtdEtn, diacyl PtdEtn, and plasmalogen PtdEtn, respectively. The content of each individual molecular species listed in column 3 was determined through ratiometric comparison of the ion peak intensity with that of the internal standard after ^13^C de-isotoping. The data represent X±SD of at least four separate animals. The notation m∶n represents the fatty acyl (or ether aliphatic) chain containing m carbons and n double bonds which do not include the vinyl ether double bond in plasmalogens. The numbers in the parentheses represent the relative composition of each individual molecular species of an isobaric ion. The symbols of “<” and “>” indicate that the data represent the best estimation from the analyses.

b Identification of individual pPtdEtn molecular species was performed based on the accurate mass analyses and acidic vapor treatment as described in the text. Identification of individual aPtdEtn molecular species was performed based on the accurate mass analyses, the paired rule, and the information of the identified pPtdEtn counterparts as discussed in the text. Identification of individual dPtdEtn molecular species was conducted based on accurate mass analyses. The abundance of each of the paired dPtdEtn molecular species cannot be accurately determined at the current stage of lipidomic technology.

If a QqQ mass spectrometer instead of a high mass accuracy/high resolution instrument was used to analyze the molecular ions corresponding to PtdEtn molecular species in general and to pPtdEtn molecular species in particular, three approaches were employed. First, we analyzed the diluted lipid extracts using negative-ion ESI/MS after treating the dried film of each lipid solution with acidic vapor (Figure 6B). The mass spectrum clearly shows the disappearance or substantial reductions in peak intensities of many ions which correspond to pPtdEtn molecular species (compare Figure 6B and 6A). The unchanged ion peaks predominantly correspond to dPtdEtn molecular species, while the residual ion peaks are those of either isomeric aPtdEtn molecular species or isobaric dPtdEtn (e.g., those containing a FA with an odd-numbered carbons) to pPtdEtn molecular species. Next, we profiled the diluted lipid extracts by using precursor-ion scanning of 196 Th (i.e., phosphoethanolamine) to further confirm the molecular ions corresponding to the pPtdEtn molecular species since these ions were enhanced (Figure 6C) due to differential fragmentation pathways as previously described [24]. Collectively, from these measurements, molecular ions corresponding to pPtdEtn and aPtdEtn were definitively identified (Table 2).

Finally, we employed a derivatization approach where the PtdEtn molecular species were tagged by addition of Fmoc and analyzed in the negative-ion mode (Figure 6D) as previously described [5], [16]. Neutral loss scanning of 222.2 u (corresponding to the Fmoc moiety) (Figure 6E) allowed us (1) to definitively identify the PtdEtn molecular ions in the mass spectrum presented in Figure 6A; (2) to eliminate overlap of PtdEtn molecular species with other molecular species from different lipid classes as determined by shotgun lipidomics; and (3) to detect the presence of very low-abundance PtdEtn molecular species. Therefore, all molecular ions corresponding to PtdEtn were determined and tabulated (Table 2). The ether aliphatic and acyl chain identities of these identified PtdEtn molecular species were similarly determined by multi-dimensional MS analyses of Fmoc-PtdEtn using a QqQ mass spectrometer as previously described [18]–[20].

### Identification of plasmalogen molecular species in combination with the paired rule facilitates the identification of alkyl-acyl phospholipid molecular species by shotgun lipidomics

It is well known that the biosynthesis of pPtdEtn molecular species occurs through their alkyl-acyl phospholipid precursors (i.e., aPtdEtn) which undergo C-1 desaturation of the alkyl chain by a desaturase activity in the endoplasmic reticulum [1]. Notably, the content of pPtdEtn molecular species in almost all mammalian tissues are considerably higher (>10 times in most cases) than those of their aPtdEtn precursors [2], [6]. Therefore, the identified paired rule for pPtdEtn molecular species is also valid for the aPtdEtn subclass of phospholipids. Moreover, since the levels of pPtdEtn molecular species in mammalian samples are always much higher than those of their corresponding aPtdEtn precursors, the identified pPtdEtn molecular species and their abundance facilitate identification of the corresponding aPtdEtn.

For example, the ion at *m/z* 772.5 contained a 20∶4 carboxylate as determined by CID analysis of bovine heart PtdEtn (Figure 3A) leading to the identification of 20∶4-20∶4 aPtdEtn. Since plasmalogens were present at this *m/z* along with the 20∶4 carboxylate, the presence of 20∶4-20∶4 aPtdEtn was inferred. This identification was substantiated by the presence of a pair of 20∶4-20∶4/20∶4-20∶4 pPtdEtn molecular species at *m/z* 770.51 (Table 1) of which the 20∶4-20∶4/20∶4-20∶4 aPtdEtn served as the metabolic precursor. Since the abundance of an aPtdEtn precursor was approximately 10 mol% or less than that of its corresponding pPtdEtn counterpart, it was anticipated that the observed 20∶4 FA carboxylate could also result from a 20∶3-20∶4 pPtdEtn species or its isomer, 20∶4-20∶3 pPtdEtn. However, the 20∶3 FA carboxylate could not be detected likely due to its extremely low abundance beyond the limits of detection using the current technology. Similarly, the presence of the paired 18∶1-22∶6/22∶6-18∶1 and 18∶2-22∶5/22∶5-18∶2 and an unpaired 20∶3-20∶4 pPtdEtn molecular species at *m/z* 772.53 facilitated the identification of the paired 18∶1-22∶6/22∶6-18∶1 and 18∶2-22∶5/22∶5-18∶2 and an unpaired 20∶3-20∶4 aPtdEtn molecular species at *m/z* 774.54 as it would be obligatory that the metabolic precursor be present (Table 1). It was found that the majority of relatively abundant aPtdEtn molecular species present in mouse cerebellar extracts were paired (25 pairs), leading to the prediction that other pairs of aPtdEtn molecular species were also likely present, but below the level of detectability due to their extremely low abundance (Table 2).

### Differential profiles of ether lipid aliphatic chains are present in comparison to non-ether lipid fatty acyl chains representative of subclass specific enzymatic selectivity during biosynthesis/remodeling

**Figure 7:**
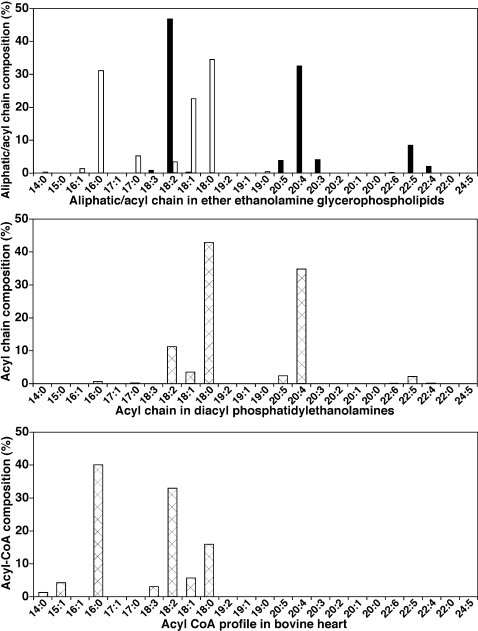
Comparison of representative aliphatic or acyl chain profiles in different lipid domains of bovine heart. The profiles of both aliphatic chains (open column in Panel A) and fatty acyl chains (closed column in Panel A) of bovine heart ether-linked ethanolamine glycerophospholipids (PtdEtn) were derived from individual molecular species listed in Table 1. The fatty acyl chain composition of bovine heart diacyl PtdEtn (Panel B) was also calculated from the identified individual molecular species as listed in Table 1. The profile of acyl-CoA in bovine heart (Panel C) was re-plotted from previously published data [28].

**Figure 8:**
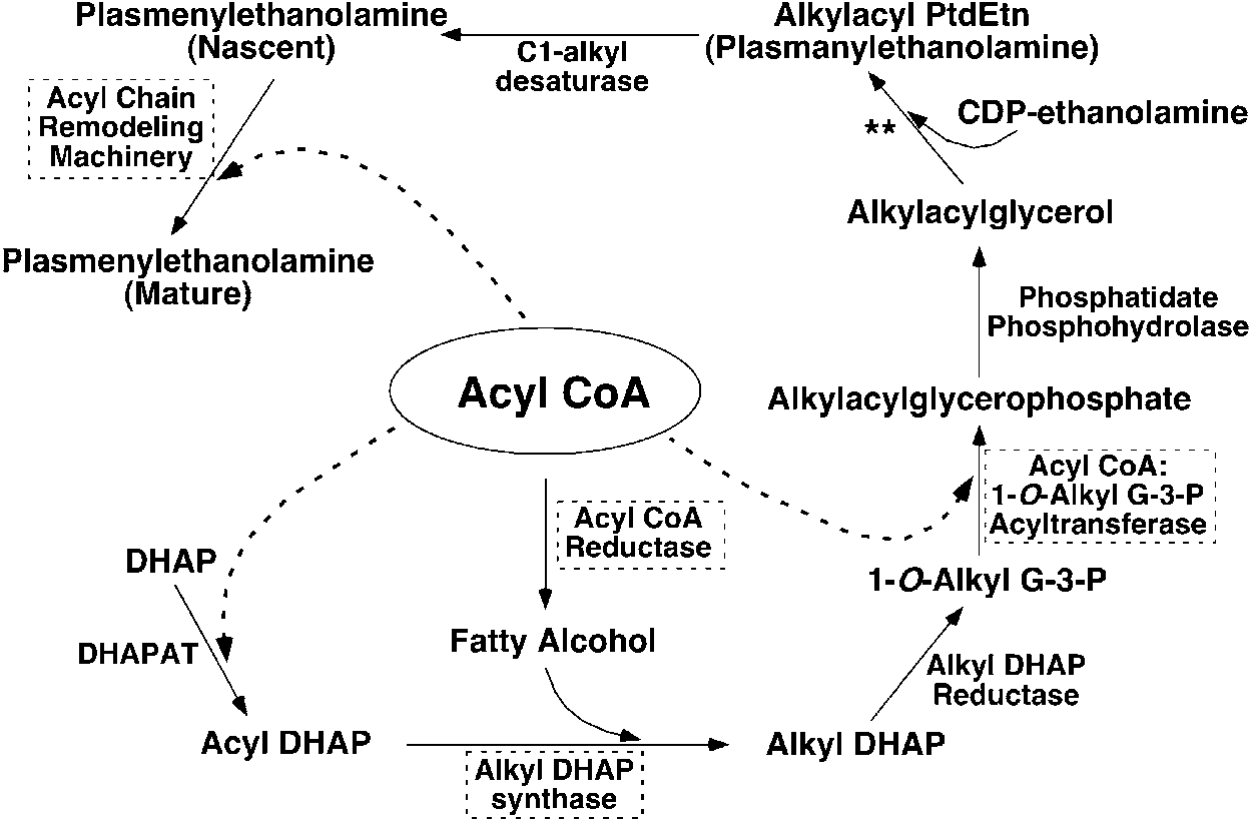
Pathways involved in the biosynthesis of plasmenylethanolamine. The enzymes that may be involved in non-selective utilization of acyl CoA pool are highlighted with broken-lined frames. ** CDP-ethanolamine: 1-*O*-alkyl-2-acyl-*sn*-glycerol ethanolamine phosphotransferase.

To further uncover the abundant, but cryptic, biochemical information present in the acyl chain composition of cellular lipids, the obligatory diversity in aliphatic chain regiospecificity predicted by the paired rule in different phospholipid molecular species was exploited. The composition of *O*-alkyl or *O*-alkenyl (*sn*-1) chains in ether PtdEtn molecular species present in bovine heart lipid extracts demonstrated that 16∶0 (31.1 mol%), 18∶0 (34.6 mol%), and 18∶1 (22.7 mol%) moieties were predominant with smaller amounts of 17∶0, 18∶2, 16∶1, 19∶0, and 14∶0 chains (in order of decreasing abundance). Other less abundant aliphatic chains collectively accounted for only 0.5 mol% of the total bovine heart ether PtdEtn. This molecular species profile was very different from that of the acyl CoA pool in bovine heart (Figure 7C). This difference clearly indicates the selective activities of the acyl-CoA reductase and the alkyl DHAP synthase which are involved in the synthesis of the obligatory precursor of ether lipids, alkyl DHAP (Figure 8).

Analyses of the fatty acyl chains derived from ether PtdEtn molecular species present in bovine heart showed a very different profile from that of either the fatty aliphatic chains of ether PtdEtn (Figure 7A) or fatty acyl-CoAs determined from the same organ [28] (Figure 7C). The fatty acyl chains of bovine heart ether PtdEtn were predominantly 18∶2 FA (46.9 mol%) and 20∶4 FA (32.6 mol%) with lesser amounts of 22∶5, 20∶3, 20∶5, 22∶4, and 18∶3 FA (8.5, 4.2, 3.9, 2.1, and 0.9 mol%, respectively). The remainder of *sn*-2 FA chains accounted for approximately 0.9 mol% of the total fatty acyl chains in ether PtdEtn. In contrast to the predominantly 16 or 18 carbon saturated or monounsaturated fatty aliphatic chains present at the *sn*-1 position of ether PtdEtn, those occupying the *sn*-2 position were essentially all polyunsaturated and the majority contained 20 or 22 carbons (Figure 7A). This fatty acyl chain profile resulted from either a selective acyl-CoA acyltransferase activity or subsequent remodeling or both (Figure 8).

Lipidomics at its current stage is unable to accurately discriminate the *sn*-1 and *sn*-2 acyl moieties present in diacyl phospholipid species, although approximate assessments of regiospecificity can be obtained from the ratios of fragment ions as previously described [24]. We summarized the overall composition of FA chains in the dPtdEtn subclass of bovine heart PtdEtn without determination of their regiospecific positions (Figure 7B). Analyses of these dPtdEtn-derived FA chains demonstrated a very different profile (Figure 7B) from either that of ether PtdEtn aliphatic/acyl chains (Figure 7A) or fatty acyl-CoAs in bovine heart (Figure 7C). The FA chains of dPtdEtn in bovine heart were predominantly 18∶0 FA (42.9 mol%) and 20∶4 FA (34.9 mol%) followed by 18∶2, 18∶1, 20∶5, 22∶5, 16∶0, and 17∶0 FA (11.4, 3.8, 2.6, 2.3, 0.8, and 0.4 mol%, respectively). The remainder accounted for 0.9 mol% of the total FA chains in bovine heart dPtdEtn. Like the FA chain profile of ether PtdEtn, this profile of FA chains derived from dPtdEtn resulted from either a selective acyl-CoA acyltransferase activity or subsequent remodeling or both (Figure 8). Although it is conceivable that the majority of the saturated FA chains were present at *sn*-1 whereas polyunsaturated FA chains were located at *sn*-2 of dPtdEtn, such a distribution is considerably different from those of ether PtdEtn (compare Figure 7B to Figure 7A).

## Discussion

The current study utilizes high mass accuracy/high resolution mass spectrometry (i.e., an LTQ-Orbitrap mass spectrometer) in conjunction with tandem mass spectrometry to greatly extend the quantitative and qualitative analyses of the ether lipid-containing lipidome in multiple organs and species. Through utilization of synthetic reagents, chemical covalent modification, and accurate mass determinations, we identified “the paired rule” of ether-containing phospholipid molecular species. This rule states that any individual ether-containing PtdEtn molecular species A′-B (where the ′ indicates the vinyl ether linkage of the aliphatic chain A at *sn-*1 and B denotes the *sn-2* acyl chain) is necessarily paired with a corresponding isomer B′-A (where B′ is the vinyl ether linkage and A is the ester) (Figure 1). The biochemical basis underlying this rule is the lack of absolute specificity in the enzymes involved in the *de novo* synthesis and remodeling of ether PtdEtn molecular species, resulting in the presence of varying amounts of the corresponding isomers depending on the relative specificity of the involved synthetic enzymes for an individual FA chain. Accordingly, this study predicts and demonstrates an inter-relationship between corresponding sets (A′-B and B′-A) of ether-containing phospholipid molecular species, greatly increases the number of known ether-containing phospholipid molecular species present and predicts the structural composition of additional ether lipid molecular species in the extremely low abundance regime.

The origination of this paired rule of ether-containing phospholipid molecular species is embedded in the biosynthetic pathway of lipids where a common substrate pool, i.e., acyl-CoA is used in multiple steps as a source of both fatty aliphatic and FA chains used in the synthesis of these phospholipids (Figure 8). Specifically, acyl-CoA is utilized by DHAP acyltransferase to synthesize the phospholipid (both diacyl and ether linked) precursor acyl DHAP in the presence of DHAP. This common pool of acyl-CoA also serves as a source of fatty alcohol through reduction mediated by NADPH-acyl-CoA reductase [1], [2]. These fatty alcohol species react with acyl DHAP through catalysis by alkyl DHAP synthase to generate alkyl DHAP which is further reduced by an alkyl DHAP reductase activity to yield 1-*O*-alkyl-glycero-3-phosphate. Acylation of 1-*O*-alkyl-glycero-3-phosphate with acyl-CoA results in alkyl-acyl glycerophosphate which leads to the final syntheses of aPtdEtn and aPtdCho, both of which are desaturated to yield pPtdEtn and pPtdCho. In theory, if any of the enzymatic activities of acyl-CoA reductase, alkyl DHAP synthase, alkyl DHAP reductase, and acyl-CoA/1-*O*-alkyl-glycero-3-phosphate acyltransferase as mentioned above is completely selective to certain fatty acyl-CoA or fatty alcohol molecular species (Figure 8) [1], [2], the final ether-containing phospholipid molecular species should be limited to specific aliphatic/acyl chains. However, the results of this study indicate that although ether aliphatic chains in PtdEtn pool were restricted to largely 16∶0, 18∶0, and 18∶1 species, much lower abundant molecular species corresponding to those predicted by the paired rule were routinely observed. It should also be pointed out that the pairing distributions are a complex function of diet, substrate availability, intracellular organelle compartmentation, and substrate-enzyme interactions at the active site. Furthermore, it is anticipated that the paired rule is also valid for ether PtdCho molecular species since they are generated from an identical pool of precursors, i.e., alkyl-acyl glycerophosphate.

The identification of “the paired rule” for ether-containing species implies that other corollaries of this rule exist in lipid biology. First, although the paired rule has been validated through identification of “matched sets” of ether-containing phospholipids, the limited regiospecificity of intracellular phospholipases renders it likely that additional pairing is present in diacyl phospholipid pools. This is further substantiated by the known isomerization of alpha hydroxyl alcohols which likely occur *in vivo*. Since the physical properties of a given pair of A-B and B-A diacyl phospholipids are separate and distinct, we speculate that such pairing may facilitate the appropriate molecular dynamics and development of specific molecular scaffolds within membranes. However, absolute proof of this hypothesis awaits the development of new lipidomic techniques to provide absolute discrimination of *sn*-1 *vs*. *sn*-2 regiospecificity.

The results of this study are the first to directly demonstrate the existence of large numbers of ether lipids present in a cellular lipidome at the limits of detectability using current lipidomic technology. Due to the limited selectivity and/or specificity of enzymes that are involved in lipid biosynthesis and remodeling, all available acyl-CoA molecular species can be incorporated into a given lipid class to some degree. Thus, the numbers of lipid molecular species present in a cellular lipidome are now recognized to be a function of detectability with their isomeric counterparts implied and/or observed in high sensitivity analyses.

Furthermore, the present results establishing the paired rule of ether-containing phospholipids will greatly aid identification of the moieties in cellular lipidomes through specifically targeting predicted molecular species during mass spectrometric analyses. This rule has defined criteria for the identification of individual molecular species of both plasmalogen and plasmanyl subclasses and for discrimination between plasmalogen and alkyl-acyl isomers. For example, in theory, the aPtdEtn precursor of each identified pPtdEtn molecular species should exist in the lipid extracts of the samples. These and other similar conceptualizations will advance lipidomics analyses to identify a new level of potentially valuable biomarkers. Although some predictable limitations due to the current technology are still present, it is clear that as the sensitivity of mass spectrometers increases these moieties will provide independent biomarkers which reflect a wide variety of turnover rates within cells. The power of this newly-identified paired rule of ether-containing phospholipid molecular species will greatly aid identification of extremely low abundance molecular species and provide new biomarkers of health and disease.

Finally, through the perspective of the paired rule, the selectivities and/or activities of enzymes that are involved in plasmalogen and plasmanyl phospholipid biosynthesis and/or remodeling during different pathophysiological states in an intact cell can be determined. Unlike previous studies in which only a limited number of abundant ether-containing phospholipids could be quantified, profiling plasmalogen and plasmanyl molecular species on a large scale as proposed herein (i.e., through exploiting high mass accuracy/resolution mass spectrometry) should dramatically enhance multiparametric assessments of the disease states of interest or their response to therapy.
